# Oral cancer patient’s profile and time to treatment initiation in the public health system in Rio de Janeiro, Brazil

**DOI:** 10.1186/s12913-021-06131-x

**Published:** 2021-02-15

**Authors:** Manoela Garcia Dias da Conceição, Isabel Cristina Martins Emmerick, Ana Claudia Figueiró, Vera Lucia Luiza

**Affiliations:** 1 Public health Post-Graduation Program National School of Public Health Sergio Arouca, Oswaldo Cruz Foundation (PPGSP/ENSP/FIOCUZ), 1480 Rua Leopoldo Bulhoes, Manguinhos, RJ, ZC 21041-210 Rio de Janeiro, Brazil; 2 Division of Thoracic Surgery, Department of Surgery, UMass Memorial Healthcare/University of Massachusetts Medical School, 67 Belmont Street #201, Massachusetts 01605 Worcester, USA; 3 Department of Collective Health, Instituto Aggeu Magalhães Institute, Oswaldo Cruz Foundation (SDC/IAM/Fiocruz), Campus da UFPE - Av. Prof. Moraes Rego, s/n - Cidade Universitária, Recife - PE, ZC 50670-420 Recife, Brazil; 4 Department of Medicines and Pharmaceutical Services Policies, National School of Public Health Sergio Arouca, Oswaldo Cruz Foundation (NAF/ENSP/FIOCUZ), 1480 Rua Leopoldo Bulhoes, Manguinhos, ZC 21041-210 Rio de Janeiro, Brazil

**Keywords:** Oral health, Mouth neoplasms, Primary Health Care, Specialized health care

## Abstract

**Background:**

This paper aims to describe the profile of oral cancer (OC) patients, their risk classification and identify the time between screening and treatment initiation in Rio de Janeiro Municipality.

**Method:**

Data were obtained from the healthcare Regulation System (SISREG) regarding the period January 2013 to September 2015. Descriptive, bivariate and multivariate analysis were performed identifying the factors associates with a diagnosis of OC as well as the time to treatment initiation (TTI) differences between groups.

**Results:**

From 3,862 individuals with a potential OC lesion, 6.9 % had OC diagnosis. OC patients were 62.3 y.o. (mean), 64.7 % male, 36.1 % were white and 62.5 % of the records received a red/yellow estimated risk classification. Being older, male, white and receiving a high-risk classification was associated with having an OC diagnosis. OC TTI was in average 59.1 days and median of 50 days significantly higher than non-OC individuals (*p* = 0.007). TTI was higher for individuals older than 60 years old, male, and white individuals and for risk classification red and yellow, nevertheless while in average none of these differences were statistically significant, the median of individuals classified as low risk was significantly (*p* = 0.044) lower than those with high risk.

**Conclusions:**

Time to treatment initiation (TTI) was higher for OC patients related to non OC. Despite OC confirmed was associated with risk at screening classified as urgent or emergent, a high percentage of OC patients had their risk classified for elective care when specialized care was requested.

## Background

Oral health is part of general health and essential to people’s well-being. Issues with oral health can have as consequences: chronic orofacial pain, cancer of the mouth and pharynx, changes in the soft tissues of the mouth, congenital disabilities, or other conditions that affect the craniofacial complex [1], leading to psychological distress. It has been associated with alcoholism, use of tobacco products [2] and social exclusion. Among its determinants, deficient schooling, low income, unemployment, and difficulty in accessing assistance services as well as deficit in self-care are identified [3, 4].

According to data from the National Cancer Institute (INCA) for Brazil, in the triennium 2020–2022 there were an estimated 625 thousands new cases per year [5] and 225 thousands deaths in 2018 [6]. Almost half of the incidence of Oral Cancer (OC) occurs in the Southeast region [6], where Rio de Janeiro Municipality is located. Nationwide there were 11,180 OC cases in the male population and around 4010 in the female population for the 2020–2022 triennium, being the fifth most prevalent among the first [5]. Although the evolution in the prevalence estimate of OC is discreet, this disease is a worldwide public health problem due to its high morbidity and mortality [7].

The vulnerability of OC subjects makes it difficult to provide adequate dental care, either because of their socioeconomic status, challenges in accessing health services, or because they do not seek health services [3]. When not treated on time, it is significantly mutilating, causing damage to these patients’ physical and psychological aspects and directly interfering with the quality of life [8].

However, if diagnosed early, OC has a good prognosis, with the average five-year survival rate in stages I and II being 77.3 %, but 32.2 % in stages III and IV [9]. As an early diagnosis of OC is uncommon, with 65 to 85 % of cases diagnosed in an advanced stage, the likelihood of cure is reduced [4, 10–12].

 In Brazil, Primary Health Care (PHC) is supposed to have an active role regarding oral health and its actions include the promotion of oral health and prevention, care, and rehabilitation. In cases of greater complexity, it must be able to act in an articulated and swift manner with the Specialized Dental Clinics (SDCs), which corresponds to secondary care. When appropriate, such as OC diagnosis, rapid treatment initiation in a cancer center is essential. Since 2012, there is in place a federal law [12] stipulating that in the event of suspected malignant neoplasia, the diagnostic confirmation test must be carried out within 30 days after medical request and specific treatment must be started within 60 days after the positive cancer diagnosis.

The care trajectory consists of patients’ path in the health care network comprising the use of health care resources from the onset of the problem to its outcome [13, 14]. It includes making appointments in oral health, the time of return to perform referrals, performing biopsies and obtaining test results, and continuity of care after starting treatment for OC [15], which must act according to a regulation system of health care delivery. The timely care is one of the components of access to the health system. In this study, it was expressed in terms of the time to treatment initiation (TTI).

Rio de Janeiro is a 6.6 million inhabitants municipality located in Southeast region. In 2009 a comprehensive plan for PHC expansion and reorganization was implemented. In addition to a broad set of governmental health facilities comprising all levels of health care, National Institute of Cancer (INCA), the main reference center for cancer care and research in the country, is located in this city. Despite of its relevance, TTI studies are scarce in Brazil and the few [16–19] were performed in limited populations. This is the first study that evaluate time for treatment initiation in the Rio de Janeiro municipality, that considered all the susceptible population.

 This study aims to describe the profile of patients with suspect and confirmed oral cancer, associated factors and time to treatment initiation (TTI) in public health system in Rio de Janeiro municipality, Brazil, from January 2013 to September 2015.

## Methods

Data were obtained from the healthcare Regulation System (SISREG) regarding the period from January 2013 to September 2015. This study was part from a broader one, a PhD dissertation that had the general aim of evaluating the quality of the care given to patients with oral cancer (OC) in the city of Rio de Janeiro considering the dimensions of access and effectiveness of primary and secondary health care delivery [20].

### Setting

Brazil has a National Health Policy Regulation since 2008 comprising three aspects: health system, health care and access to health care, mediated by an information system. SISREG was the patient flow regulation systems used in the Brazilian Unified Health System (SUS) until 2015, when was replaced by Rio de Janeiro State Regulation System (SER) [21]. This change the data structure and storage, which made the linkage of pre and post changes complex and unworthy for the purpose of the source study. Therefore, the analysis used the complete data prior to this administrative change.

This study concerns to Rio de Janeiro municipality, a 6.6 million inhabitants (population at 2012) Brazilian City [22] and a complete health services offer, comprising all health care levels.

**Fig. 1:**
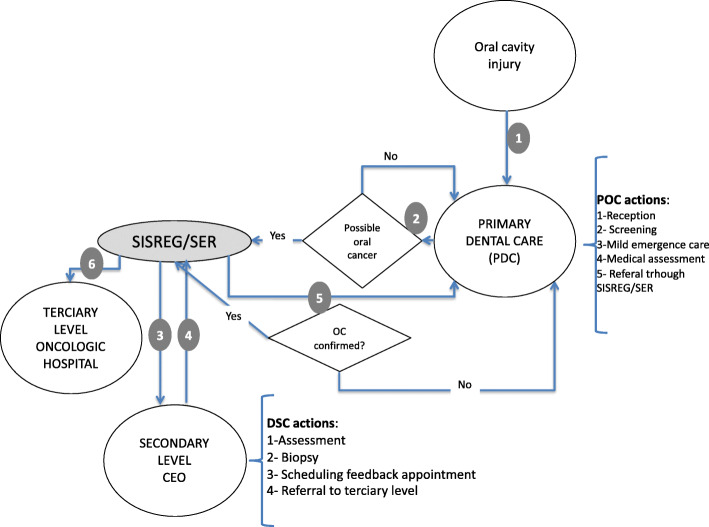
Recommended flow of care for lesions suspected of oral cancer. OC = Oral Cancer; PDC = Primary Dental Care; SDC = Specialized Dental Clinics

Oral Care counts on a regionalized and hierarchical services network, offered in PHC, secondary, high complexity and urgency / emergency health facilities [7]. Secondary OC is provided in Specialized Dental Clinics (SDC) (Centro de Especialidades Odontológicas (CEO), in Portuguese), that offer Endodontics, Periodontics, Minor surgery, Stomatology, Orthodontics and Prostheses care as well as care for patients with special needs [7]. The recommended patient flow is presented in Fig. 1.

To initiate care, patients must look for their reference PHC facility which is also supposed to offer health promotion, and disease prevention activities. In case of specialized care need, the patient must be referred to a SDC, preferably also close to his home, through SISREG. If a more specialized care is required, e.g., cancer treatment, the patient is referred also through SISREG.

The database contained all referral records analyzed in the regulation system during the interest period by following medical specialties: general head and neck, head and neck oncology, and dentistry / stomatology.

### Variables

The main outcome variable was ‘having oral cancer’. The following factors were investigated: age (average and categories), excluding individuals under 15 years old; race (white vs. non-white) and first risk stratification categorized as high risk (red or yellow) and low risk (green or blue) and TTI. Risk categories are blue - elective care; green - not urgent; yellow – urgent; and red – emergent. In Brazil, the variable race is related with skin color/ethnicity and the recommendation for its collection is self-declaration by the interviewee. Despite of the name “race” have been considered obsolete in the literature, many activists argue that is important to value this variable in order to evidence disparities in the context of access to care and treatment. The time to treatment initiation was defined as the time between the first request date recorded in that database and the last execution date, what indicates the time between the first appointment and the moment that the individual was seen in his / her definitive treatment facility (start of OC treatment).

Each encounter referred was recorded in SISREG, thus the same individual can have multiple entries in the system and each record receive a risk classification. The overall risk analysis was performed by encounter. To perform the bivariate and multivariate statistical analyzes it was considered the risk evaluation attributed to the patient in the first encounter.

### Analysis

For the first main outcome, the presence of oral cancer, a descriptive analysis was performed considering the distribution of age, sex and race, as well as bivariate and multivariate analysis. The difference of proportions was expressed by the odds ratio, aiming to identify differences in the demographics and risk determination between cancer and non-cancer patients.

 The second outcome was the TTI, and this analysis comprised a descriptive analysis (mean and median) by presence of oral cancer diagnosis. For those with a positive diagnosis of oral cancer, it was performed the non-parametric test of median difference and T-test of the mean to verify if the TTI was different between groups by the age, sex and race. As the distribution of the variable waiting time was heterogeneous, mean and median were very different it was made the option of statistically test the T-test (mean) and the non-parametric tests for median differences.

All statistical analyses were performed at 95 % of confidence interval using SPSS V.22.

### Ethical issues

 The source study was approved by three Ethical Research Committees, the first from academic institution, the National School of Public Health Sergio Arouca ERC (CAAE 59009416.6.0000.5240), the second from the National Institute of Cancer ERC (CAAE 59009416.6.3001.5274), the hospital where patients were sampled for the face-to-fac interview (data not included in this paper) and the third belonging from the Rio de Janeiro Municipality Health Secretariat ERC (CAAE 59009416.6.3002.5279).

## Results

It was found a total of 3,862 individuals with a potential OC lesion in the SISREG. A total of 266 (6.9 %) individuals were confirmed as OC. The average age was 52.3 years old for those with no cancer and 62.3 years old in the OC group and there were no cases of oral cancer under 15 years old. Males’ percentage was higher among those with OC (64.7 %) and the percentage of white individuals was 36.1 % and 27.5 % among patients with and with no OC respectively (Table 1).

**Table 1:** Demographic characteristics by oral cancer diagnosis, SISREG. Rio de Janeiro Municipality, January 2013 to September 2015

Variables	Oral cancer					
	No (***N***=3596)		Yes (***N***=266)		Total (***N***=3862)	
	N	%	N	%	N	%
**Age (mean in years)**	52.3		62.3		53	
**Age (range of years)**						
<1	3	0.1	0	-	3	0.1
>=1 e <15	240	6.7	0	-	240	6.2
>=15 e <60	1820	50.6	104	39.1	1924	49.8
>=60 e <85	1446	40.2	151	56.8	1597	41.4
>=85	87	2.4	11	4.1	98	2.5
**Sex**						
female	2263	62.9	94	35.3	2357	61.0
male	1333	37.1	172	64.7	1505	39.0
**Race**						
asian	425	11.8	22	8.3	447	11.6
white	988	27.5	96	36.1	1084	28.1
indigenous	2	0.1	0	-	2	0.1
brown	771	21.4	47	17.7	818	21.2
black	350	9.7	26	9.8	376	9.7
No information	1060	29.5	75	28.2	1135	29.4

From the total 4.764 records, 64.3 % were classified as blue and 15.1 % as red. It was found 27.2 % records as red risk among the male while 9.2 % among the female. Among those with OC 45.2 % were classified as red risk, while this percentage was 12.9 among those with no OC. Among those records diagnosed as OC and risk classified as red the majority (77.1 %) were male (Table 2). The majority of the patients that received a diagnosis of cancer also received a red risk stratification.

**Table 2:** Distribution of estimated risk of SISREG records by selected variables by diagnosed oral cancer, SISREG. Rio de Janeiro Municipality, January 2013 to September 2015

Variable			Oral cancer				Total	
			No		Yes			
			#	%	#	%	#	%
**Total**	-	-	4439	100.0	325	100.0	4764	100.0
**Estimated risk**		BLUE - elective care	2941	66.3	121	37.2	3062	64.3
		GREEN - not urgent	213	4.8	1	0.31	214	4.5
		YELLOW - urgent	711	16.0	56	17.23	767	16.1
		RED - emergent	574	12.9	147	45.23	721	15.1
**Sex**	BLUE - elective care	Female	1474	65.4	47	46.1	1521	3.1
		Male	781	34.6	55	53.9	836	6.6
	GREEN - not urgent	Female	133	71.9	1	100.0	134	0.7
		Male	52	28.1	0	0.0	52	-
	YELLOW - urgent	Female	391	60.3	19	42.2	410	4.6
		Male	257	39.7	26	57.8	283	9.2
	RED - emergent	Female	265	52.2	27	22.9	292	9.2
		Male	243	47.8	91	77.1	334	27.2

*FH* Federal Hospital, *SH* State level hospital, *UH* University Hospital

The likelihood (adjusted OR) of having cancer and receiving a risk score yellow or red was 3.0 [95 %CI 2.2–4.3]. Males, individuals 60 years old and greater, and white were more likely to receive a diagnosis of cancer, with OR respectively of (OR 2.7 [95 %CI 2.0–3.8]). (1.8 95 % CI 1.3–2.5) and (OR = 1.4 [95 %CI 1.0–1.9) (Table 3).

**Table 3:** Study population demographic characteristics associated with the oral cancer diagnosis, SISREG. Rio de Janeiro Municipality, January 2013 to September 2015

Variables (dummy)		Cancer Oral # (percentage within Cancer Oral)		Total	*P*-value Gross OR (95 % CI)	*P*-value Adjusted OR (95 % CI)
		No	Yes			
Risk	Blue or Green	2440 (67.9 %)	103 (38.7 %)	2543 (65.8 %)	*p* = 0.000	*p* = 0.000
	Yellow or Red	1156 (32.1 %)	163 (61.3 %)	1319 (34.2 %)	3.3 (2.6–4.3)	3.0 (2.2-4.0)
**Total**		**3596 (100.0 %)**	**266 (100.0 %)**	**3862 (100.0 %)**		
Sex	Female	2263 (62.9 %)	94 (35.3 %)	2357 (61.0 %)	*p* = 0.000	*p* = 0.000
	Male	1333 (37.1 %)	172 (64.7 %)	1505 (39.0 %)	3.1 (2.4–4.0)	2.7 (2.0-3.8)
**Total**		**3596 (100.0 %)**	**266 (100.0 %)**	**3862 (100.0 %)**		
Age	>=15 e < 60	1820_a_ (54.3 %)	104 (39.1 %)	1924 (53.2 %)	*p* = 0.000	*p* = 0.000
	>=60	1533_a_ (45.7 %)	162 (60.9 %)	1695 (46.8 %)	1.8 (1.4–2.4)	1.8 (1.3–2.5)
**Total**		**3353 (100.0 %)**	**266 (100.0 %)**	**3619 (100.0 %)**		
Race	Not white	1548 (61.0 %)	95 (49.7 %)	1643 (60.2 %)	*p* = 0.003	*p* = 0.050
	white	988 _(_39.0 %)	96 (50.3 %)	1084 (39.8 %)	1.6 (1.6–2.2)	1.4 (1.0 -1.9)
**Total**		**2536 (100.0 %)**	**191 (100.0 %)**	**2727 (100.0 %)**		

The average TTI, which is time from the first request and the appointment for those with no OC was 47.1 days and median of 23 days. Meanwhile, for patients with OC confirmed, the average TTI was 59.1 days and median of 50.5 days until being admitted in a cancer hospital and the difference was significant (*p* = 0.007) (Table 4). The TTI average was higher for individuals older than 60 years old, male, and white individuals and for risk classification red and yellow.

**Table 4:** Mean and median of time to treatment initiation by diagnosed oral cancer, age, sex, race and risk, SISREG. Rio de Janeiro Municipality, January 2013 to September 2015

Variables	N	Mean	SD	***P***-value	Median	***P***-value
Non-Oral Cancer	3596	47,1	70,7	**0,007**	23,0	**0,000**
Oral Cancer	266	59,1	60,7		50,5	
Age Group						
>=15 e <60	104	53,8	53,3	0,254	39,0	0,379
>=60	162	62,5	64,9		56,5	
Race						
Non-white	95	59,3	60,1	0,405	46,0	0,168
White	96	67,3	72,1		61,5	
Sex						
Male	172	62,0	59,6	0,294	56,0	0,158
Female	94	53,8	62,6		35,5	
Risk						
Blue or Green	103	56,4	77,4	0,571	31,0	**0,044**
Yellow or Red	163	60,8	47,4		56,0	

Statistically significant *p*-values 95% confidence interval are indicated in bold

It was noticed that the TTI medians also followed the same patters as the means, nevertheless with greater difference. While in average none of these differences were statistically significant, the median of individuals classified as high risk was significantly (*p* = 0.044) higher than those with low risk.

## Discussion

Oral Cancer (OC) had a prevalence of 6.9 % in this study, with greater proportions in the older, white and males. The risk classification was the main important factor in the likelihood of having OC diagnosis. Half of the patients with oral cancer had treatment initiation within 51 days and the median of individuals classified as high risk was significantly higher than those with low risk. No gender and race disparities in terms of TTI were found.

Since 2004, with the expansion of the National Oral Health Policy (PNSB) [3], Brazil has implemented its strategies for prevention, early diagnosis, and control of OC. The PNSB implementation requires easy access to oral health services through the Family Health Strategy (ESF) and the Oral Health Teams as PHC interventions [23].

Articulated actions offered in a timely and resolutive way can prevent the late diagnosis and improve patients’ prognostics. In this sense, the OC approach must include the regular screening for early detection; assessment of oral lesions (active search, home visits, specific campaigns); monitoring of suspected cases; referral services for confirmed cases; and the establishment of partnerships for prevention, diagnosis, treatment, and recovery with universities and other organizations [24].

Public health programs are expected to provide adequate responses to the health problems for which they are intended and evaluated for implementation, access, and outcomes, guiding decision-making [25]. Thus, it is worth asking about the offer of oral health care within the scope of the PNSB in primary, secondary and specialized health services and how patients have covered the services they need.

There is a higher likelihood of receiving a diagnosis of OC for individuals over 60 years old, males, white, which corroborates with results of other studies regarding the profile of patients and location of lesions [26, 27].

When OC diagnosed patients were referred through the SISREG system, the average time to start cancer treatment (average of 50 days and median of 59 days) is within the federal legal time period of 60 days [28]. Nevertheless, the Rio de Janeiro municipality health regulation has a more restrictive TTI rule that establishes that patients diagnosed with cancer must be referred by PHC, via SISREG, for specialized care. Other studies that evaluated TTI in Brazil found it as 1–3 months [18], 23 days mean (1-116 days range) [17] and 71.1 days (1-142 days range) [19]. Nevertheless, they all used small hospital-based samples. A study from INCA found a TTI below 60 days 43.8 % and 56.2 for male and female patients, respectively in the Southeast Region [29]. Studies carried out in foreign countries found an average TTI of 30 days [30] and greater than 46 days [31] for 25 % of treated patients, a time considered worrying for the disease outcome.

When his risk is classified as high (red), this requires priority scheduling of up to 30 days. Yellow, scheduling up to 90 days, green and blue, scheduling up to 180 days or more, respectively, was found not to adhere [32]. As well established in the literature, the TTI can be decisive for the progress and incurability of the disease [33, 34], and preventable delays should be avoided.

Among the causes for the time elapsed between the first registration of the patient in the regulation system and the beginning of treatment, one can assume the flow instituted by the health system since the PHC. For cases registered as having the highest risk for OC (red and yellow risk), the time to start treatment was longer than for the others. Serra et al. also found an inadequate organization of referral and counter-referral activities, with many of the patients not being referred by the official system, which produces double entry into the sector, resulting in losses or delays in some visits [35]. The opposite, such as decentralization and regionalization of assistance for cancer treatment, facilitate patient access, with an increase in the number of hospitalizations in some locations [36].

Another relevant factor is the difficulty in identifying a suspected lesion of these tumors, being most often diagnosed when its size exceeds 2 cm [37]. This situation and other factors can lead to a delay in diagnosis, as indicated by Costa-Jr and Serra [28]: few and nonspecific symptoms, patients’ lack of knowledge about the disease, little familiarity of general practitioners who work in primary care with the diagnosis of cancer and difficulty in accessing the health system [38]. The late diagnosis is reflected in the most frequent treatments at the referral hospital, radiation therapy associated with surgery, knowing that the best prognosis is referred to as only surgical treatment [38].

As limitations it is important to mention that the regulation system does not include staging and other clinical information that would be helpful and could support and inform policy changes needed in the regulation and referral. Also, the SISREG is a secondary database and as such, can contain misclassifications of cases. Finally, only registered individuals that had their treatment through SUS were analyzed.

## Conclusions

In this study, it was found that being male, white, and older than 60 increased the likelihood of having a diagnosis of oral cancer. The time to initial treatment was higher for those with confirmed OC diagnosis; nevertheless, TTI is within the time expectation established by Law in the National Health System and there were no disparities regarding age, race and gender. The risk classification system seems to not be a factor for speeding up the treatment, furthermore the median TTI of individuals with high-risk classification was higher and reasons for that should be clarified in future studies.

## Data Availability

The datasets generated and/or analyzed during the current study is not publicly available due to its sensitive information (PHI) Protected Health Information and sharing is not allowed by the owner, but are available from the corresponding author on reasonable request.

## Abbreviations

CI: Confidence Interval
ERC: Ethical Research Committee
ESF: Family Health Strategy
INCA: National Cancer Institute
OC: oral cancer
OR: Odds Ratio
p: p value
PHC: Primary Health Care
PNSB: National Oral Health Policy
SDC: Specialized Dental Clinics (*Centro de Especialidades Odontológicas – CEO*, in Portuguese)
SER: Rio de Janeiro State Regulation System
SISREG: healthcare Regulation System
SUS: Unified Health System
TTI: time to treatment initiation
y.o.: years old
